# Ventilator-associated pneumonia or not? Contemporary diagnosis.

**DOI:** 10.3201/eid0702.010209

**Published:** 2001

**Authors:** C G Mayhall

**Affiliations:** University of Texas Medical Branch, Galveston, Texas, USA. cmayhall@utmb.edu

## Abstract

Ventilator-associated pneumonia (VAP) is pneumonia in patients who have been on mechanical ventilation for > or =48 hours. VAP is most accurately diagnosed by quantitative culture and microscopy examination of lower respiratory tract secretions, which are best obtained by bronchoscopically directed techniques such as the protected specimen brush and bronchoalveolar lavage. These techniques have acceptable repeatability, and interpretation of results is unaffected by antibiotics administered concurrently for infection at extrapulmonary sites as long as antimicrobial therapy has not been changed for <72 hours before bronchoscopy.


### Ventilator-Associated Pneumonia or Not? Contemporary Diagnosis

**C. Glen Mayhall**
University of Texas Medical Branch, Galveston, Texas, USA

Ventilator-associated pneumonia (VAP) is pneumonia in patients who have been on mechanical ventilation for ≥48 hours. VAP is most accurately diagnosed by quantitative culture and microscopy examination of lower respiratory tract secretions, which are best obtained by bronchoscopically directed techniques such as the protected specimen brush and bronchoalveolar lavage. These techniques have acceptable repeatability, and interpretation of results is unaffected by antibiotics administered concurrently for infection at extrapulmonary sites as long as antimicrobial therapy has not been changed for <72 hours before bronchoscopy.

Ventilator-associated pneumonia (VAP) is defined as nosocomial pneumonia in a patient on mechanical ventilatory support (by endotracheal tube or tracheostomy) for ≥48 hours. For many years, VAP has been diagnosed by the clinical criteria published by Johanson et al. in 1972, which include the appearance of a new or progressive pulmonary infiltrate, fever, leukocytosis, and purulent tracheobronchial secretions (1); however, these criteria are nonspecific (2). In the mechanically ventilated patient, fever may be caused by a drug reaction, extrapulmonary infection, blood transfusion, or extrapulmonary inflammation. Pulmonary infiltrates may be due to pulmonary hemorrhage, chemical aspiration, pleural effusion, congestive heart failure, or tumor. Both fever and pulmonary infiltrates occur in the fibroproliferation of late acute respiratory distress syndrome, atelectasis, and pulmonary embolism, as well as in VAP. Cultures of tracheal aspirates are not very useful in establishing the cause of VAP (2). Although such cultures are highly sensitive, their specificity is low even when they are cultured quantitatively (3).

VAP can be accurately diagnosed by any one of several standard criteria: histopathologic examination of lung tissue obtained by open lung biopsy, rapid cavitation of a pulmonary infiltrate in the absence of cancer or tuberculosis, positive pleural fluid culture, same species with same antibiogram isolated from blood and respiratory secretions without another identifiable source of bacteremia, and histopathologic examination of lung tissue at autopsy (4). However, these criteria are based on invasive procedures for obtaining lung tissue or on uncommon manifestations or complications of VAP. Given the invasive nature of lung biopsy and the infrequent occurrence of other manifestations used as standard criteria, another approach is needed for the definitive diagnosis of VAP. In 1979, a fiberoptic bronchoscopic technique was introduced for obtaining uncontaminated lower respiratory tract secretions, which were cultured quantitatively (5). The causative microorganisms were recovered at $\geq 10^3$ CFU/mL from six patients with clinical evidence of lower respiratory tract infection.

Address for correspondence: C. Glen Mayhall, Division of Infectious Diseases, 301 University Boulevard, Route 0435, University of Texas Medical Branch, Galveston, TX 77555-0435; fax: 409-772-6527; e-mail: cmayhall@utmb.edu

In 1987, a correlation was observed between pneumonia and $\geq 10^5$ CFU/mL in bronchoalveolar lavage (BAL) fluid (6,7). Kahn and Jones noted that BAL fluid with $\geq 10^5$ CFU/mL and ≤1% squamous epithelial cells had 100% sensitivity and specificity for the diagnosis of bacterial pneumonia.

Two bronchoscopic techniques have been introduced for the accurate diagnosis of VAP in the absence of standard criteria. The protected specimen brush (PSB) collects 0.001 mL of lower respiratory tract secretions and has a diagnostic threshold of $\geq 10^3$ CFU/mL (8). BAL, an unprotected technique, samples approximately one million alveoli and has a diagnostic threshold of $\geq 10^4$ CFU/mL (8). A protected BAL technique with a balloon-tipped catheter has also been described (9). Detection of ≥5% of neutrophils or macrophages with intracellular organisms on a Wright-Giemsa stain of a smear of cytocentrifuged BAL fluid is also diagnostic of VAP (10).

### Bronchoscopically Directed Techniques for Diagnosis of VAP

The accuracy of quantitative culture and microscopic examination of lower respiratory tract secretions for the diagnosis of VAP was validated by Chastre et al. (10,11), who compared the results of quantitatively cultured lower respiratory tract secretions with those of culture and histopathologic examination of simultaneously obtained lung tissue. In the first study, quantitative culture of secretions obtained by PSB was compared with histopathologic examination and quantitative culture of lung tissue (11). Of six patients with pneumonia confirmed by histologic criteria, all had at least one microorganism obtained at a concentration of $\geq 10^4$ CFU/g of lung tissue. Compared with the results of histologic examination and quantitative culture of lung tissue, quantitative culture of secretions obtained by PSB using a diagnostic threshold of $\geq 10^3$ CFU/mL had a sensitivity of 100%, specificity of 60%, positive predictive value of 43%, and negative predictive value of 100%.

In the second study, the results of PSB, BAL, and ≥5% intracellular organisms were compared with simultaneously obtained lung tissue (Table) (10). Patients were included in

Table. Quantitative cultures and microscopy examination of lower respiratory tract secretions in the diagnosis of ventilator-associated pneumonia[a]

| Diagnostic techniques | Sensitivity | Specificity | Positive predictive value | Negative predictive value |
|---|---|---|---|---|
| PSB[b] cultures ($\geq 10^3$ CFU/mL) | 82% | 89% | 90% | 89% |
| BAL cultures ($\geq 10^4$ CFU/mL) | 91% | 78% | 83% | 87% |
| Microscopic examination of BAL fluid ($\geq$5% intracellular organisms) | 91% | 89% | 91% | 89% |

[a]From ref 10.
[b]PSB = protected specimen brush; BAL = bronchoalveolar lavage.

the study only if they had never had pneumonia or had acquired it during the terminal phase of their illness. Bronchoscopy was performed within 1 hour after death, while mechanical ventilation was continued and PSB and BAL samples were taken. Immediately after bronchoscopy, a left thoracotomy was performed, and lung tissue specimens were taken from the areas of lung where the bronchoscopic samples had been obtained. All but two patients had been receiving antibiotics before death, but antibiotic therapy had not been changed for $\geq$3 days. All lung segments judged to have moderate to severe pneumonia by histologic criteria yielded $\geq 10^4$ CFU/g of tissue.

Four other published studies have concluded that bronchoscopically directed techniques were not more accurate for diagnosis of VAP than clinical and X-ray criteria combined with cultures of tracheal aspirates (12-15). In one study, quantitative cultures of lower respiratory tract secretions obtained by PSB and BAL were compared with quantitative culture and histopathologic examination of lung tissue taken from the same areas sampled by PSB and BAL (12). These investigators used $\geq 10^3$ CFU/g of lung tissue as a threshold for positive cultures of lung tissue; in addition, patients were enrolled at any time during mechanical ventilation, so that pulmonary infiltrates could have been included from earlier pneumonia or current pneumonia with bacteria previously eradicated from some foci and still present in other areas of the lung. When multiple inflammatory foci of varying ages are present in the lungs, histopathologic examination and culture of lung tissue may not correlate with results of quantitative cultures of simultaneously obtained lower respiratory tract secretions.

Other investigators compared the results of quantitative culture and microscopic examination of lower respiratory tract secretions obtained by PSB and BAL with histopathologic examination of lungs at autopsy performed within 3 days of bronchoscopic sampling of the lower airways (13). Specificity and positive predictive values for cultures of secretions collected by PSB and BAL were comparable with those observed by Chastre et al. (10,11); however, substantially lower sensitivities of 57.8% and 47.3% and negative predictive values of 51% and 48% were observed for PSB and BAL, respectively. These discrepant findings may be due to the study design, in which sampling of lower airways and examination of lung tissue were separated by up to 3 days, the areas from which PSB and BAL samples were taken could not be precisely matched with the same areas examined histopathologically, and lung tissue could not be cultured because lungs were examined at autopsy.

In a comparative study, quantitative culture and microscopic examination of lower respiratory tract secretions were compared with histopathologic examination and quantitative culture of lung tissue obtained from the same area of the lung from which samples of secretions were taken (14). These investigators observed 70% specificity and 65% positive predictive value for bronchoscopically guided PSB and 63% sensitivity and 79% negative predictive value for bronchoscopically guided BAL. These patients were on mechanical ventilation for a mean of 14 days and a median of 8 days and could have acquired one or more episodes of pneumonia at any time while on mechanical ventilation. In addition, 38 of 39 patients received antibacterial or antifungal therapy in the 48 hours before death. However, duration of therapy or change of antimicrobial therapy in the 72 hours before death was not stated. If antimicrobial therapy had been changed, bacteria susceptible to the newly instituted antimicrobial agents might not have been recovered on culture of respiratory secretions and lung tissue of patients who had histopathologic evidence of pneumonia.

In another study, the results of quantitative culture and microscopic examination of lower respiratory tract secretions were compared with histopathologic examination and quantitative culture of simultaneously obtained lung tissue in 25 patients on mechanical ventilation immediately after death (15). Whether patients on antibiotic therapy at the time of death had any changes in therapy in the 72 hours before death or whether they had earlier episodes of VAP before the episode of pneumonia diagnosed at the time of death was not stated. In addition, these workers used $\geq 10^3$ CFU/g of tissue rather than $\geq 10^4$ CFU/g as the threshold for positive lung cultures, which may account for the lower sensitivity, specificity, and positive and negative predictive values for quantitative culture of secretions obtained by bronchoscopically directed PSB and BAL.

### Nonbronchoscopically Directed (Blind) Diagnostic Techniques

Because of the invasive nature and cost of bronchoscopy, investigators have evaluated other techniques for collecting lower respiratory tract secretions. These nonbronchoscopic techniques involve passage of a catheter or telescoping catheters through the endotracheal tube with advancement to a wedged position in the lung. Samples may be taken by telescoping catheters containing a brush (blind PSB) (16-18), aspiration of secretions into a distally wedged catheter (19,20), or BAL through a distally wedged catheter (21-24). BAL may be performed by using a balloon-tipped catheter with the balloon inflated after the catheter has been advanced to the wedged position (protected BAL) (21), by using telescoping catheters (22,24), or by placing a catheter into the wedged position with a guide wire (23).

Although nonbronchoscopic or blind techniques for obtaining lower respiratory tract secretions appear promising, additional validation studies are needed before these techniques are widely adopted and can be used in place of bronchoscopically directed sampling techniques. Studies of nonbronchoscopic sampling techniques have recently been reviewed (25). Another indication of the need for further study of the nonbronchoscopic sampling techniques is the absence of standardized diagnostic thresholds for quantitative culture of lower respiratory tract specimens obtained by these techniques.

### Quantitative Cultures To Predict VAP Onset and Monitor Therapy

To predict the onset of VAP in patients with adult respiratory distress syndrome (ARDS), Delclaux et al. used quantitative culture of lower respiratory tract secretions obtained blindly by passing a plugged telescopic catheter through the endotracheal tube (26). They observed that in 16 of 18 patients lower respiratory tract colonization ($<10^3$ CFU/mL) evolved to pneumonia within 2 to 6 days. Colonizing microorganisms were the same as those that caused subsequent pneumonia. The 89% positive predictive value of lower respiratory tract colonization for pneumonia further substantiates the accuracy of quantitative culture of lower respiratory tract secretions for the diagnosis of VAP.

Quantitative culture of lower respiratory tract secretions can also be used to monitor the progress of antimicrobial therapy for VAP. Montravers and co-workers diagnosed VAP in 76 patients by using quantitative culture of lower respiratory tract secretions obtained through bronchoscopically directed PSB and recovered 135 isolates at $\geq 10^3$ CFU/mL (27). When a second PSB was performed by bronchoscopy 3 days after start of therapy, 126 (93%) of the initial 135 isolates were not recovered by the second PSB, 7 (5.2%) were recovered at $<10^3$ CFU/mL, and 2 (1.5%) were still present at $\geq 10^3$ CFU/mL. The last two isolates were the only bacteria resistant to initial treatment because of errors in selection of antibiotics. Thus, results of quantitative cultures of respiratory secretions obtained by repeat PSB were consistent with the antimicrobial susceptibilities of isolates obtained by the first PSB. The authors noted that when follow-up PSB cultures were negative, the patients' conditions improved. This study further supports the accuracy of quantitative culture of lower respiratory tract secretions for the diagnosis of VAP.

### Repeatability of PSB and BAL

Repeatability, which is defined as the variation in repeated measurements of the same quantity (28), is one measure of the accuracy of a technique in diagnosing the diseases(s) for which it was developed. Marquette and associates performed a study in which a single investigator performed bronchoscopy on 22 patients with suspected VAP (28). At each bronchoscopy, five successive PSB samples were taken from the same area of the lung. All PSB specimens were cultured quantitatively by the same technologist. In each patient, all five PSB procedures identified exactly the same microorganisms. In 59% of the patients, there was more than a 1-log variation in quantitative culture of the five PSB specimens; in 3 (13.6%) of the 22 patients, quantitative culture results were spread out on both sides of the $10^3$ CFU/mL breakpoint. Thus, in spite of the substantial variability of the quantitative cultures, all five PSB procedures for 19 (86.4%) of 22 patients gave results on the same side of the breakpoint, indicating acceptable repeatability.

The repeatability of BAL was assessed in a study in which two BALs were performed in the same lobe 30 minutes apart in 44 patients (29). The bronchoscope was sterilized between procedures in each patient. The investigators observed that both BALs yielded negative results in 28 patients and that the same microorganism was recovered from both BALs in 14 of 16 patients. Thus, 40 of 44 pairs of BAL samples yielded the same results, for a repeatability of 90.9%. Results of duplicate BALs for 4 (25%) of the 16 patients with positive cultures were spread out on both sides of the $10^4$ CFU/mL diagnostic threshold. Overall, BAL appears to have an acceptable (75%) level of repeatability in patients with positive cultures. Additional studies of the repeatability of PSB and BAL are needed.

### Antibiotics and Diagnosis of VAP by Quantitative Culture of Lower Respiratory Tract Secretions

When patients with pneumonia are receiving antimicrobial agents at the time lower respiratory tract secretions are obtained for diagnosis of VAP, cultures may be negative, and concentrations of bacteria may be below the diagnostic threshold. Such uncertainty about the interpretation of culture results from patients on antibiotics has prompted study of the effect of antibiotics on the diagnosis of VAP. Timsit and co-workers assessed the impact of antimicrobial therapy on the diagnosis of VAP by collecting lower respiratory tract secretions by bronchoscopically directed PSB and BAL from patients with suspected VAP (30). Ninety-six patients had not received antimicrobial agents for $\geq 3$ days before bronchoscopy, while 65 patients had been on antibiotics for $\geq 3$ days at the time PSB and BAL samples were obtained. Sensitivity and specificity did not differ for PSB, BAL, and percentage of intracellular organisms in patients receiving and not receiving antibiotics. The authors concluded that when patients acquire pneumonia while on antibiotics for infections at extrapulmonary sites, the microorganisms are resistant to these antibiotics and the diagnostic yields of PSB and BAL are unaffected.

Souweine et al. (31) confirmed and extended the observations of Timsit and co-workers. In 63 episodes of suspected VAP, 12 patients had received no antibiotics in the 4 days before bronchoscopy, 31 had been treated with antibiotics for >72 hours, and 20 had begun antibiotics or had their antibiotic regimen modified within the 24 hours before bronchoscopy. The diagnosis of VAP was made by bronchoscopically directed PSB, BAL, and microscopic examination for intracellular organisms. The sensitivity for the diagnosis of VAP by percentage of intracellular organisms did not differ in the three groups. Nor did the sensitivity of PSB and BAL differ in the group not receiving antibiotics and the group receiving antibiotics for >72 hours. In the group of patients with initiation or change of antibiotics in the 24 hours before bronchoscopy, the sensitivity of PSB and BAL decreased substantially but was restored by reducing the threshold for PSB to $10^2$ CFU/mL and for BAL to $10^3$ CFU/mL. These studies suggest that the sensitivity of PSB and BAL for the diagnosis of VAP is unchanged in patients who acquire VAP while on antibiotics for >72 hours for treatment of an extrapulmonary infection. Therefore, for such patients lower respiratory tract secretions should be obtained for quantitative culture and microscopic examination before any changes are made in antimicrobial therapy.

### Diagnosis of VAP in Patients with ARDS

VAP is more common in patients with ARDS than in those with other causes of respiratory failure (26,32,33); it occurs later and is caused by more resistant microorganisms. The diagnosis of VAP is more difficult in such patients because ARDS and VAP have very similar clinical manifestations. Chastre et al. observed no significant differences in temperature, leukocyte count, $Pao_2/Fio_2$ ratio, or radiologic score in patients with ARDS with and without VAP (32). Since clinical criteria for VAP lack both sensitivity and specificity in patients with ARDS, microbiologic data are thought to play a prominent role in the diagnosis of VAP that complicates ARDS (26). In a study of the use of bronchoscopically directed BAL to diagnose VAP in patients with ARDS, bronchoscopic findings modified antibiotic therapy in 91% of patients with positive BAL cultures and prevented the use of new antibiotics in 54% of patients with insignificant growth (33). Given the severity of illness of patients with ARDS, particularly when complicated by VAP, and the great difficulty in differentiating VAP from ARDS on clinical and radiographic grounds, the most effective approach to diagnosis of VAP in patients with ARDS is quantitative culture and microscopic examination of lower respiratory tract secretions.

### Data Quality in the Diagnosis of VAP

Quantitative culture and microscopic examination of lower respiratory tract secretions are most effective when attention is paid to the quality of specimens from the lower respiratory tract (8,34,35). The following practices are recommended: 1) Antibiotics should not be started or changed until after lower respiratory tract secretions have been obtained. 2) When bronchoscopically directed techniques are used, secretions should not be suctioned nor anesthetic injected through the working channel of the bronchoscope. 3) Less than 10% return of instilled fluid during BAL probably represents inadequate sampling of the lower respiratory tract. 4) When lower respiratory tract sampling is performed by PSB, the brush must be placed into exactly 1 mL of fluid. 5) Specimens should be delivered immediately to the laboratory. 6) Fewer than 10 cells per field at a magnification of 500x in fluid obtained by PSB probably represents an inadequate sample; resampling should be considered. 7) The presence of >1% epithelial cells indicates an unreliable sample; additional samples should be obtained.

In conclusion, in the absence of gold standard criteria for the diagnosis of VAP, the diagnostic test of choice is quantitative culture and microscopic examination of lower respiratory tract secretions. This approach provides the most accurate diagnosis of VAP and identification of the causative microorganism(s), can predict the onset of VAP and provide the identity and susceptibility of the causative microorganism(s) at the time clinical manifestations of VAP appear, can be used to assess the cause of therapy failure, provides the most effective modality for diagnosis of VAP that complicates ARDS, minimizes misclassification of cases of VAP for studies on the epidemiology of VAP, and minimizes the selective pressure for development of resistant microorganisms. Whether this approach to the diagnosis of VAP has an effect on outcome and reduces deaths is yet to be determined.

Dr. Mayhall is Professor of Internal Medicine, University of Texas Medical Branch at Galveston, and Hospital Epidemiologist, University of Texas Medical Branch Hospitals and Clinics. His research interests are in hospital-acquired infections, including antimicrobial-drug resistance, nosocomial infections in obstetrics, and intravascular device-associated infections.

### References

1. Johanson WG Jr, Pierce AK, Sanford JP, Thomas GD. Nosocomial respiratory infections with gram-negative bacilli. The significance of colonization of the respiratory tract. Ann Intern Med 1972;77:701-6.
2. Meduri GU. Diagnosis of ventilator-associated pneumonia. Infect Dis Clin North Am 1993;7:295-329.
3. Jourdain B, Novara A, Joly-Guillou M-L, Dombret M-C, Calvat S, Trouillet J-L, et al. Role of quantitative cultures of endotracheal aspirates in the diagnosis of nosocomial pneumonia. Am J Respir Crit Care Med 1995;152:241-6.
4. Fagon J-Y, Chastre J, Hance AJ, Domart Y, Trouillet J-L, Gibert C. Evaluation of clinical judgment in the identification and treatment of nosocomial pneumonia in ventilated patients. Chest 1993;103:547-53.
5. Wimberley N, Faling LJ, Bartlett JG. A fiberoptic bronchoscopy technique to obtain uncontaminated lower airway secretions for bacterial culture. Am Rev Respir Dis 1979;119:337-43.
6. Thorpe JE, Baughman RP, Frame PT, Wesseler TA, Staneck JL. Bronchoalveolar lavage for diagnosing acute bacterial pneumonia. J Infect Dis 1987;155:855-61.
7. Kahn FW, Jones JM. Diagnosing bacterial respiratory infection by bronchoalveolar lavage. J Infect Dis 1987;155:862-9.
8. Meduri GU, Chastre J. The standardization of bronchoscopic techniques for ventilator-associated pneumonia. Infect Control Hosp Epidemiol 1992;13:640-9.
9. Meduri GU, Beals DH, Maijub AG, Baselski V. Protected bronchoalveolar lavage. A new bronchoscopic technique to retrieve uncontaminated distal airway secretions. Am Rev Respir Dis 1991;143:855-64.
10. Chastre J, Fagon J-Y, Bornet-Lecso M, Calvat S, Dombret M-C, Khani RA, et al. Evaluation of bronchoscopic techniques for the diagnosis of nosocomial pneumonia. Am J Respir Crit Care Med 1995;152:231-40.
11. Chastre J, Viau F, Brun P, Pierre J, Dauge M-C, Bouchama A, et al. Prospective evaluation of the protected specimen brush for the diagnosis of pulmonary infections in ventilated patients. Am Rev Respir Dis 1984;130:924-9.
12. Torres A, El-Ebiary M, Padró L, Gonzalez J, de la Bellacasa JP, Ramirez J, et al. Validation of different techniques for the diagnosis of ventilator-associated pneumonia. Comparison with immediate postmortem pulmonary biopsy. Am J Respir Crit Care Med 1994;149:324-31.
13. Marquette CH, Copin M-C, Wallet F, Neviere R, Saulnier F, Mathieu D, et al. Diagnostic tests for pneumonia in ventilated patients: prospective evaluation of diagnostic accuracy using histology as a diagnostic gold standard. Am J Respir Crit Care Med 1995;151:1878-88.
14. Kirtland SH, Corley DE, Winterbauer RH, Springmeyer SC, Casey KR, Hampson NB, et al. The diagnosis of ventilator-associated pneumonia. A comparison of histologic, microbiologic, and clinical criteria. Chest 1997;112:445-7.
15. Fábregas N, Ewig S, Torres A, El-Ebiary M, Ramirez J, de la Bellacasa JP, et al. Clinical diagnosis of ventilator associated pneumonia revisited: comparative validation using immediate post-mortem lung biopsies. Thorax 1999;54:867-73.
16. Torres A, de la Bellacasa JP, Rodriguez-Roisin R, DeAnta MTJ, Agusti-Vidal A. Diagnostic value of telescoping plugged catheters in mechanically ventilated patients with bacterial pneumonia using the Metras catheter. Am Rev Respir Dis 1988;138:117-20.
17. Jordá R, Parras F, Ibañez J, Reina J, Bergadá J, Raurich JM. Diagnosis of nosocomial pneumonia in mechanically ventilated patients by the blind protected telescoping catheter. Intensive Care Med 1993;19:377-82.

18. Marik PE, Brown WJ. A comparison of bronchoscopic vs blind protected specimen brush sampling in patients with suspected ventilator-associated pneumonia. Chest 1995;108:203-7.
19. Papazian L, Martin C, Albanese J, Saux P, Charrel J, Gouin F. Comparison of two methods of bacteriologic sampling of the lower respiratory tract: a study in ventilated patients with nosocomial bronchopneumonia. Crit Care Med 1989;17:461-4.
20. Pham LH, Brun-Buisson C, Legrand P, Rauss A, Verra F, Brochard L, et al. Diagnosis of nosocomial pneumonia in mechanically ventilated patients. Comparison of a plugged telescoping catheter with the protected specimen brush. Am Rev Respir Dis 1991;143:1055-61.
21. Gaussorgues P, Piperno D, Bachmann P, Boyer F, Jean G, Gérard M, et al. Comparison of nonbronchoscopic bronchoalveolar lavage to open lung biopsy for the bacteriologic diagnosis of pulmonary infections in mechanically ventilated patients. Intensive Care Med 1989;15:94-8.
22. Rouby J-J, Rossignon M-D, Nicolas M-H, de Lassale EM, Cristin S, Grosset J, et al. A prospective study of protected bronchoalveolar lavage in the diagnosis of nosocomial pneumonia. Anesthesiology 1989;71:679-85.
23. Pugin J, Auckenthaler R, Mili N, Janssens J-P, Lew PD, Suter PM. Diagnosis of ventilator-associated pneumonia by bacteriologic analysis of bronchoscopic and nonbronchoscopic “blind” bronchoalveolar lavage fluid. Am Rev Respir Dis 1991;143:1121-9.
24. Kollef MH, Bock KR, Richards RD, Hearns ML. The safety and diagnostic accuracy of minibronchoalveolar lavage in patients with suspected ventilator-associated pneumonia. Ann Intern Med 1995;122:743-8.
25. Mayhall CG. Nosocomial pneumonia. Diagnosis and prevention. Infect Dis Clin North Am 1997;11:427-57.
26. Delclaux C, Roupie E, Blot F, Brochard L, Lemaire F, Brun-Buisson C. Lower respiratory tract colonization and infection during severe acute respiratory distress syndrome. Incidence and diagnosis. Am J Respir Crit Care Med 1997;156:1092-8.
27. Montravers P, Fagon J-Y, Chastre J, Lecso M, Dombret MC, Trouillet J-L, et al. Follow-up protected specimen brushes to assess treatment in nosocomial pneumonia. Am Rev Respir Dis 1993;147:38-44.
28. Marquette CH, Herengt F, Mathieu D, Saulnier F, Courcol R, Ramon P. Diagnosis of pneumonia in mechanically ventilated patients. Repeatability of the protected specimen brush. Am Rev Respir Dis 1993;147:211-4.
29. Gerbeaux P, Ledoray V, Boussuges A, Molenat F, Jean P, Sainty J-M. Diagnosis of nosocomial pneumonia in mechanically ventilated patients. Repeatability of the bronchoalveolar lavage. Am J Respir Crit Care Med 1998;157:76-80.
30. Timsit J-F, Misset B, Renaud B, Goldstein FW, Carlet J. Effect of previous antimicrobial therapy on the accuracy of the main procedures used to diagnose nosocomial pneumonia in patients who are using ventilation. Chest 1995;108:1036-40.
31. Souweine B, Verber B, Bedos JP, Gachot B, Dombret MC, Regnier B, et al. Diagnostic accuracy of protected specimen brush and bronchoalveolar lavage in nosocomial pneumonia: impact of previous antimicrobial treatments. Crit Care Med 1998;26:236-44.
32. Chastre J, Trouillet JL, Vuagnat A, Joly-Guillou ML, Clavier H, Dombret MC, et al. Nosocomial pneumonia in patients with acute respiratory distress syndrome. Am J Respir Crit Care Med 1998;157:1165-72.
33. Meduri GN, Reddy RC, Stanley T, El-Zeky F. Pneumonia in acute respiratory distress syndrome. A prospective evaluation of bilateral bronchoscopic sampling. Am J Respir Crit Care Med 1998;158:870-5.
34. Gallego M, Rello J. Diagnostic testing for ventilator-associated pneumonia. Clin Chest Med 1999;20:671-9.
35. Mertens AH, Nagler JM, Galdermans DI, Slabbynck HR, Weise B, Coolen D. Quality assessment of protected specimen brush samples by microscopic cell count. Am J Respir Crit Care Med 1998;157:1240-3.
